# Effect of Psychological Factors on Credit Risk: A Case Study of the Microlending Service in Mongolia

**DOI:** 10.3390/bs11040047

**Published:** 2021-04-05

**Authors:** Mandukhai Ganbat, Erdenebileg Batbaatar, Ganzul Bazarragchaa, Togtuunaa Ider, Enkhjargalan Gantumur, Lkhamsuren Dashkhorol, Khosgarig Altantsatsralt, Mandakhbayar Nemekh, Erdenebaatar Dashdondog, Oyun-Erdene Namsrai

**Affiliations:** 1 Department of Research and Development, Optimal N Max LLC, Bogd Javzandamba Street, Khan-Uul District, LS Plaza 801, Ulaanbaatar 17011, Mongolia; mandukhai@optimal.mn (M.G.); ganzul@optimal.mn (G.B.); togtuun@optimal.mn (T.I.); enkhjargalan@optimal.mn (E.G.); lkhamsuren@optimal.mn (L.D.); khosgarig@optimal.mn (K.A.); mandakhbayar@optimal.mn (M.N.); 2 Department of Information and Computer Sciences, School of Engineering and Applied Sciences, National University of Mongolia, Ikh surguuliin gudamj 3, Sukhbaatar District, P.O. Box-46A/600, Ulaanbaatar 14201, Mongolia; erdenebileg11@gmail.com; 3 Department of Physics, School of Arts and Sciences, National University of Mongolia, Ikh surguuliin gudamj 1, Sukhbaatar District, P.O. Box-46A/600, Ulaanbaatar 14201, Mongolia

**Keywords:** behavior economics, psychological factors, determinants, the psychology of personality, psychometric analysis, economic factors, credit risk, the responsible borrower, borrower, personality trait, loan repayment, indebtedness

## Abstract

This paper determined the predefining factors of loan repayment behavior based on psychological and behavioral economics theories. The purpose of this research is to identify whether an individual’s credit risk can be predicted based on psychometric tests measuring areas of psychological factors such as effective economic decision-making, self-control, conscientiousness, selflessness and a giving attitude, neuroticism, and attitude toward money. In addition, we compared the psychological indicators to the financial indicators, and different age and gender groups, to assess whether the former can predict loan default prospects. This research covered the psychometric test results, financial information, and loan default information of 1118 borrowers from loan-issuing applications on mobile phones. We validated the questionnaire using confirmatory factor analysis (CFA) and achieved an overall Cronbach’s alpha reliability coefficient greater than 0.90 (α = 0.937). We applied the empirical data to construct prediction models using logistic regression. Logistic regression was employed to estimate the parameters of a logistic model. The outcome indicates that positive results from the psychometric testing of effective financial decision-making, self-control, conscientiousness, selflessness and a giving attitude, and attitude toward money enable individuals’ debt access possibilities. On the other hand, one of the variables—neuroticism—was determined to be insignificant. Finally, the model only used psychological variables proven to have significant default predictability, and psychological variables and psychometric credit scoring offer the best prediction capacities.

## 1. Introduction

In practice, most financial institutions make their credit decisions on the financial and demographic characteristics of their customers and do not take into account personal factors. In the opinion of the interactionist perspective in psychology, individuals’ behaviors are not only explained by situational and economic factors but also individuals’ needs and personality traits. Based on this approach, it can be seen that individuals’ personality traits can be a key indicator of an individual’s loan repayment behavior [1].

A psychometric methodology based on psychological factors can identify the loan repayment behavior of microloan service customers. Globally, FinTech companies and the other loan service providers are experimenting and developing technology of psychometric credit score systems. These psychometric tests that recognize the financial behavior of individuals are implemented in over 50 countries worldwide, capable of predicting the financial decisions of individuals with 91% accuracy [2]. The benefit of this development is characterized by increased accessibility to the loan service for those capable of paying their loan but lacking a prior credit score and with limited ability to obtain credit. This development crucially influences poverty reduction by providing loan opportunities to billions of people unable to obtain a loan due to substandard financial conditions and the absence of credit history.

The commercial banks and non-banking financial organizations of Mongolia have traditionally determined the creditworthiness of the individual based on limited resources such as individual income and credit history [3]. On the other hand, as of the first quarter of 2019, the total volume of the non-performing debts reached 89.1 billion MNT or 9.1% of the net loan [4], which demonstrates the ineffectiveness of the traditional credit assessment method, which is based on the material resources of the individual for the loan purpose, and the inability to prevent non-performing loan risks. The majority of the working-age population in Mongolia is unable to obtain loans from banks and non-banking financial institutions (NBFIs) because they are deemed not credit-worthy due to their low income and absence of loan history, collateral, or social insurance payment. Amongst each 10 salaried adults in Mongolia, 1 has a high-income level, 3 are in the medium-income category or are able to demonstrate their creditworthiness for the bank and financial services, and the remaining 6 people are in a low-income class and are unable to provide documentary evidence for credit services [5]. We believe that using psychological credit scoring increases the number of low-income but trustworthy customers.

Research in this area has not yet been conducted in Mongolia. International studies show that economic and demographic indicators are widely used to predict loan repayments. In the last decade, rather than just economic and demographic factors, psychological factors consisting of personality traits, attitudes, and behaviors have also been emphasized as potential predictors of repayment behavior in debt-related studies [1].

In 2006, the Entrepreneurial Finance Lab (EFL, Massachusetts Avenue, Cambridge, Massachusetts 01238 USA) of Harvard University first studied the possibility of employing psychological methods of borrower profiling in practice [6]. The EFL developed a psychometric methodology of the borrower’s personality assessment under the three categories of personality traits, intellectual ability, and fairness [7]. The methodology developed by the EFL of Harvard focused on personality traits and fairness characteristics and leveraged these for developing an assessment methodology based on the theories of individual psychology.

This study considers six psychological determinants, highlighted in the literature and theoretical research, as the factors influencing repayment behavior: effective financial decision-making, self-control, conscientiousness, selflessness and a giving (charitable) attitude, and neuroticism. These psychological factors will be explained in detail in the theoretical background section.

In order to choose the determinants, we relied on some of the following literature and theories. The findings of previous research [1] revealed that “Rational decision making” is a prominent characteristic of regular payers, while “irrational decision making” is revealed as characteristic of irregular payers. As defined by Daniel Kahneman and Amos Tversky, the founders of behavioral economics, rational decision-making enables effective financial decision making [8]. Mahfuzur Rahman et al. (2020) found that self-control is important to avoid indebtedness [9]. In addition, conscientiousness is a protective factor in high-risk borrowing behavior, and it is associated with lower levels of unsecured borrowing [10]. The phenomenon of “attitude towards money” was studied by Russian researchers, and in their research models they included an analysis of individual psychological, personal, socio-psychological, and social factors that determine the intensity of their attitude towards money, according to different scales laid down in the methodology. Thus, the relationship between social and money attitudes and the economic activity of an individual is shown [11]. In the research on socio-psychological peculiarities and level of financial literacy of Russian debtors, Nyhus and Webley (2001) found that emotional instability as neuroticism is a positive predictor of debt [12].

This study aims to determine personality traits and psychological factors affecting debt repayment behavior and compare through the financial indicators, and the study is expected to contribute and help to fill a gap in the literature. Moreover, the findings of this research are expected to reveal repayment-related psychological assessment special to Mongolian culture, which also makes this study valuable. To meet this goal, we developed a new set of psychometric questionnaires to identify the predefined factors and tested the questionnaires at the loan issuing business entity.

Finally, the adult customers of the loan issuing organization above 18 years old, with monetary sources and experience with managing them, were surveyed with a questionnaire that consisted of three sections of demographic data, financial information, and a psychological test. The loan issuance decision was made based on how the customer responded to the selected psychological questions of the psychometric test. To ensure the quality of research, it is mandatory to have a sufficiently large dataset that contains the customer’s information at the moment of the loan application and post loan credit history. The application of the above methodologies using the stated sample enables the correlation between the borrower’s characteristics and further loan repayment behavior.

The remainder of the paper is structured as follows. Section 2 discusses the theoretical basis of the study. Section 3 describes the data samples and methodology, Section 4 presents the results, and Section 5 describes findings and discussion. Finally, Section 6 presents the conclusion, implications, and limitations.

## 2. Theoretical Background

Indebtedness is a planned and rational decision that allows intertemporal redistribution of consumption [13]. However, there is an evidence regarding cognitive bias, that our financial decisions are influenced more by our emotions, attitude, and behavioral traits than logical thinking.

To understand the financial decision-making in unpredictable conditions, throughout the development of behavioral economics for over 20 years, individual behavior and attitude have been closely observed by behavioral economists [14] based on psychological research works [15]. As defined in behavioral economics theories, system-1 - the human model of automatic thinking and system-2-the human model of mechanical thinking directly influence the decision making of humans [16]. Since cognitive bias, thoughts, and attitude are the internal factors that establish the individual, we argue that identification of the responsible borrower shall rely on the individual’s psychological study.

According to the dispositional approach, individuals have stable traits that influence their behaviors. These traits are primarily unobservable characteristics or mental states such as values, needs, or personalities. From this perspective, it can be said that personality and psychological states may have an effect on the debt-repayment behavior of an individual [1]. In addition to this, in the literature, it is argued that demographic and economic factors are not enough to explain the debt-repayment behavior of individuals. Psychological factors need to be considered for understanding and explaining these behaviors [17,18]. Moreover, psychologist Sara Hampson defined the psychological processes of the individual, such as thoughts, emotions, motivations, and perceptions, as a mechanism that develops the influence of personality traits of the individual over time [19]. It could be expected to explain the personality traits if the internal processes of the individual were well studied.

All things considered, in our survey, we distinguished the differences between responsible borrowers and high-risk borrowers with overdue loans and missed payments. Loan repayment behavior can be analyzed with a focus on their causes. That type of work is devoted to the search of predictors of debt behavior, which include, for instance, self-control and time perspective, like in the study by Webley and Nyhus (2001) [12]. However, it is not always possible to distinguish the relationship between the internal characteristics of individuals and debt accumulation. For example, Gathergood’s works (2012) [20] state that a low level of self-control leads to higher debt, as respondents with weak self-control often use short-term but expensive loans, and the increase in debt is due to low self-control, not financial literacy.

In previous studies, personality factors such as effective financial decision-making [1], self-control [9], conscientiousness [1,21], selflessness and giving (charitable) attitude, neuroticism [21], and money attitude have come out as predominant factors influencing repayment behavior.

**Figure 1:**
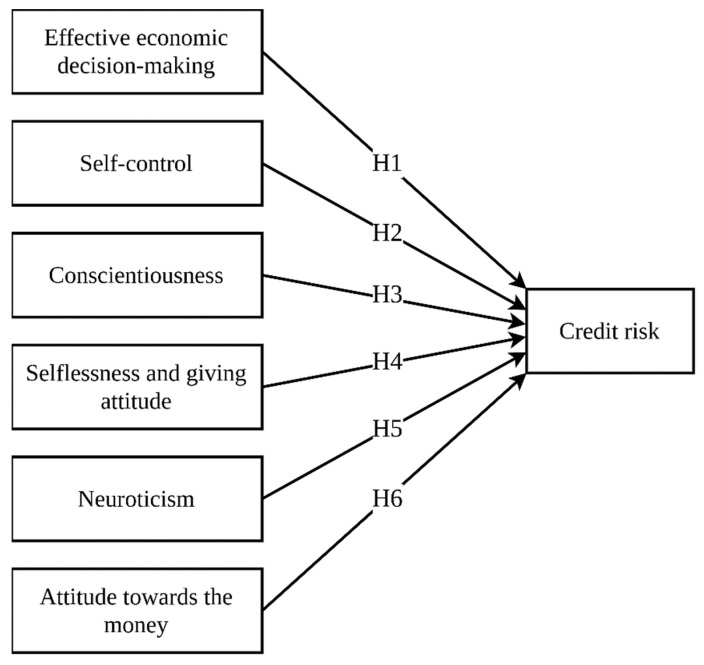
Research model and hypotheses.

Thus, based on literature about the psychology of personality, characteristics, behavior, and decision-making in particular, we selected to identify six factors to predict the borrower repayment probability and risks. Figure 1 shows the research model and the hypothesis of this study.

Moreover, according to the research conducted by the Organization for Economic Co-operation and Development in 2012, financial discipline correlates with proper financial acts, while proper financial acts are associated with the positive attitude towards money [22]. Some of the research indicators that prove the above hypothesis include the following: (1) a higher level of income and neurosis of the individual indicate they are more likely to exceed loan overdue [23]; (2) venturesome individuals are inclined to enter into indebtedness; (3) people who are aware of their savings and control of their expenses tend to spend money in a more controlled manner; (4) people who are dazed by the cashier’s bill have a higher probability of having larger debts [24].

### 2.1. Psychological Variables

#### 2.1.1. Effective Financial Decision-Making

Financial discipline means essential knowledge, understanding, skills, attitude, and action of the individual directed in making effective decisions and the content of their decision (OECD). The immediate reasons for ineffective decision making include having (1) a physiological or environmental barrier in deducing rational conclusions and (2) insufficient or incorrect information. If there is no provisional reason, the decision-making relates to the individual’s personality, self-control, and emotion. It is an important determinant of whether an individual has good personal financial behavior such as having sufficient savings for future consumption [9].

The winner of the Nobel Memorial Prize in Economic Sciences, Richard H. Thaler, has proven the direct relation of emotion and feelings to financial decision-making (nudge theory). He published a book on the effects of bias-inducing heuristics on the quality of financial decision-making.

According to Kahneman, our process of thinking contains intuitional sense and is easily affected by the heuristic approach. Thus, people tend to be overconfident in their deceived decisions [25]. The most palpable example of the emotional and short-term decision is the act of purchasing unnecessary items on a sentimental background. Under the exact same principle, the most inefficient financial decisions are made. Hence, the application of consciousness and logically overcoming the selections based on the emotional ground, or as defined by Daniel Denneth [26] using the brain mechanically, shall be advantageous. As a result, individuals will gain the capability to control the debt payment ratio and further become debt-free and allocate a certain percentage of the budget for purchasing the items they wish for. Despite the natural limits on people’s cognitive capacity and a wealth of literature on the role of bias in decision-making and behavior generally, there has been surprisingly little research relating to cognitive biases and the systematic errors in heuristics (short-cuts) we specifically use in our thinking and judgements to borrowing [10].

**Hypothesis** **1** **(H1).** *The ability to make efficient economic decisions is significant in predicting the borrower risk.*

#### 2.1.2. Self-Control

The concepts of self-control would also seem very relevant for use of consumer credit and, more particularly, consumer over-indebtedness. Generally, self-control means the ability to break away from negative habits, suppressing their anger, and overcoming emotional impulses. Concerning the financial operations, this is the psychological ground for identifying the potential over-consumption of credit cards. Studies in the theoretical literature have commonly cited self-control problems as a possible explanation for high levels of credit card borrowing [27,28,29]. Self-control also serves as a psychological factor, useful in predicting financial behavior and financial wellbeing [30]. Empirical studies on measuring self-control problems among individuals have found a negative relationship between measured self-control and the accumulation of wealth [31,32].

While most of the people with identified weak self-control tend to commit sudden and impulse purchases [33], the detailed analysis of people with overdue debt demonstrates overly emotional and extravagant behavior, unable to understand the ways to manage their finance [20].

The results of one study [20] provide convincing evidence that the lack of self-control and a low level of financial literacy are positively correlated with defaults on consumer loans and self-reports of excessive debt burden. Therefore, self-control is more significant in statistical terms and implies stronger economic effects in all specifications [34].

**Hypothesis** **2** **(H2).** *Self-control has a significant impact on timely debt payment.*

#### 2.1.3. Conscientiousness

Paying the loan on time and being able to save money and avoid impulse purchases are the characteristics of the financially disciplined individual. Amongst the Big Five personality traits, conscientiousness has a positive interrelation with financial discipline, while neuroticism has a negative correlation with financial discipline [35]. Some research findings suggest that debtors have a lower level of conscientiousness, debt avoidance, and rational debt behavior than borrowers/payers and non-borrowers [21]. Solid evidence comes from an analysis of the British Household Panel Survey, in which conscientiousness predicted lower unsecured borrowing [10].

A conscientious individual is more likely to focus on the future and able to incorporate a view for the future into their actions. Financially, they are more self-controlled, and this characteristic influences their financial discipline the most [35]. Individuals of this type of personality trait are advantageous for being responsible to themselves, comparable to being conscientious to others. Some researchers determined that conscientious individuals can accumulate savings dedicated to their health and pension [36], are less affected by the financial pressure of youthhood [37], and have a better credit history [12].

Therefore, we selected the key characteristics of conscientious individuals as a measurement indicator.

**Hypothesis** **3** **(H3).** *A conscientious personality significantly reduces the risk of having overdue credit.*

#### 2.1.4. Selflessness and Giving (Charitable) Attitude

A generous and charitable attitude means providing monetary and material support, spending time, caring, helping, motivating, and emotionally supporting others [38]. The mesolimbic pathway is sometimes referred to as the reward pathway of the brain that activates when a person helps or gives to others, similar to having food or laughing. Similar to feeling happy for any pleasant action, a person feels satisfaction after helping others [39]. In other words, charitable people are prevailingly happier with stable psychology and have a gentler approach.

We assume that people who prefer not to spend money for others are likely to have less motivation to repay loan. Nevertheless, some studies have found that people are happier when spending money on others, than on themselves, because of the higher level of endorphins produced [38]. People unable to spend money on others and that tend to hold grudges we may assume are lacking the soul to pay the loan back [38].

According to our hypothesis, generous people or people with charitable attitudes have higher intent of paying the loan back.

**Hypothesis** **4** **(H4).** *Charitable attitude and selfless personality significantly/positively affect the loan risk.*

#### 2.1.5. Neuroticism

This type of individual personality trait is explained as a negative tendency towards things and focusing on unfavorable future events [40]. Amongst the “Big Five” personality traits, neuroticism is most relevant to financial discipline and psychological stability. According to the research on interrelations between the neuroticism and individual financial management capability, neuroticism significantly impacts the tendency of spending over one’s income; individuals encounter problems of overdue utility payments and exceeded maximum credit card limits [41,42].

As defined in the psychological theories, when the individual loses their balance in stressful, anxious, fearful, and shameful situations, the brain seeks many other acts or things, such as having food, nicotine, and alcohol, and conducts improper financial maneuvers to recover and adjust the balance [43]. Hence, unnecessary purchases are made, induced by neuroticism. As identified by the other studies, highly neurotic people are less likely to request a loan compared to less neurotic people. It is induced by their fear of refusal. The study also deduced refusal probability is higher as well [44].

**Hypothesis** **5** **(H5).** *Neurosis significantly affects loan defaults.*

#### 2.1.6. Attitude towards Money

The individual’s attitude towards money is articulated by their reaction and acceptance of financial and monetary problems [45]. Psychological science of money studies the interrelations between the human decisions of spending, squandering, or saving money from their attitude, behavior, emotion, sense, value, and habit developed since their childhood [46,47], and their parents play a crucial role [48]. Altering the attitude developed in childhood is an arduous task that requires a long time [45].

To describe individual differences in the motivation for obtaining and spending money, four money attitudes have been differentiated in prior research [47,49,50]. Based on these types, the low-risk group of individuals consists of a principled individual, one who prefers to save. Risk-avoider people favor safety and circumvent risks and money worries; their fear reduces with control over their finances, and people who spend too much time on money control and budgeting belong to this category. The high-risk group includes the following: spenders—no understanding of saving and budgeting money; money monks—feel bad when in possession of a lot of money; money avoiders—try to avoid daily tasks related to money and budgeting and are afraid of thinking about money; money amassers—always wishing for money with the main target of becoming rich; bingers—tend to economize for a while, but if affected by an external stimulus, they are susceptible to shop without consideration; and risk-takers—perceive money as a source of means for adventure, excitement, and freedom. We consider studying these attitude dimensions as efficient to identify inappropriate financial maneuvers, and we use them as a measurement indicator in this study.

**Hypothesis** **6** **(H6).** *A positive attitude towards money positively affects the loan risk.*

## 3. Methodology

### 3.1. Data Collection

A total of 1118 borrowers completed a pre-interview questionnaire (all questions are provided in Supplementary File). The points for answers were calculated between the ranges −2 and 2. The surveys were conducted via a mobile application between May 2019 and May 2020 in Mongolia. The dependent variable (credit risk) of the defaulted borrower was marked as risky = 0, and the non-defaulted was marked as non-risky = 1.

### 3.2. Test Developing Steps

There are no validated psychological questionnaires to assess psychological factors impacting loan repayment behavior in Mongolia, such as effective economic decision-making (EDM), self-control (SCR), conscientiousness (CON), selflessness and giving attitude (SGA), neuroticism (NRT), and attitude towards money (ATM). For this reason, a new questionnaire has been developed through considering international economic behavioral studies and psychological works to address this issue. As shown in Table 1, experience in other countries shows that the following methodologies are used to develop new tests and questionnaires:

Based on the above methods, we developed the test in the following phases.

#### 3.2.1. Establish Expert Committee

A committee consisting of 5 psychologists and 2 economists was established. Psychologists and economists have conducted theoretical studies of the factors that affect loan repayment behavior.

#### 3.2.2. Identify Dimensionality of Construct

Theoretical research has identified that factors such as effective financial decision-making (EDM), self-control (SCR), conscientiousness (CON), selflessness and giving attitude (SGA), neuroticism (NRT), and attitude towards money (ATM) can play the main role of determining customer repayment behavior, and these factors are considered as main components of the construction.

#### 3.2.3. Item Development

In accordance with the first three steps of Phase 3 of the “Test Developing Method 2” as mentioned above, the questionnaires were developed within the framework of the 6 factors that make up the construct, and they were reviewed to see if they were consistent with the theoretical part and the factors.

#### 3.2.4. Review and Revise Initial Item Pool

An expert revision was obtained on the developed questionnaire. Necessary corrections were made based on the expert’s conclusions. The revised questionnaire itself contained 111 questions (see Supplementary File).

#### 3.2.5. Pilot Testing

The finalized questionnaire was conducted in the following two ways to test and make improvements:

1. Focus group discussion

A focus group discussion was conducted with 20 attendees who identified as non-risky (10) and risky (10) through traditional financial scoring. The purpose of the discussion was to support the reliability and clarity of the psychological questionnaire and, moreover, to reveal how Mongolian culture and certain personality traits and attitudes impact loan repayment behavior. Discussions were conducted, and all discussions were recorded by a video recorder with the permission of the participants. Participants were informed that all information they shared will be used just for academic purposes and will not be published without their permission.

The video recording (120 min) was transcripted into a written document by researchers the following day. Then, data obtained from 20 attendees were coded and analyzed by 5 different researcher-psychologists.

2. Pilot testing

The questionnaire was clarified and improved by focus group discussion results, and it was tested and interpreted with 280 responses.

#### 3.2.6. Reliability/Validity Check

In order to test the reliability of the study, we calculated Cronbach’s alpha as a measure of internal consistency of the questionnaire, which resulted in a Cronbach’s alpha = 0.937, making it possible to use the questionnaire in the future. Furthermore, we began measuring the internal consistency of each scale. Scales for effective financial decision-making (EDM), self-control (SCR), conscientiousness (CON), selflessness and giving attitude (SGA), neuroticism (NRT), and attitude towards money (ATM) showed above-average reliability (internal consistency; Cronbach’s alpha = 0.677–0.913, Table 2). Specifically, the effective economic decision-making scale, which had a value α = 0.913, was greater than 0.90, which could be considered good. The self-control and attitude towards money scales had acceptable values of alpha (ranging from 0.70 to 0.95), and the other three scales were very close to what could be considered acceptable. Once approved, the psychometric test was used in this study. See examples in Table 3, which contain hypothesis codes, questions, answers, and points for corresponding answers. All questions are provided in Supplementary File.

### 3.3. Statistics for the Sampled Data

The hypotheses discussed in the previous section are depicted by the research model presented in Figure 1. The sample was made up of 51.25% females and 48.75% males. In terms of age, the final sample comprised 15.65% aged 18–24 years, 67.44% aged 25–39 years, 15.3% aged 40–54 years, and 1.61% aged 55–75 years. Table 4 shows the statistics of the samples in detail.

### 3.4. Economic Categories

In addition, we compared the psychological variables through the well-known economic categories. The non-banking financial sector in Mongolia, with 2.3 million consumers, uses traditional methods of calculation based on individual financial analysis when issuing microloans [4]. Traditional methods of calculation take economic factors into account, such as previous credit history, income and expenditure ratio, collateral, payment of social insurance premiums, and valuations of other assets in ownership. Yet, the number of non-performing loans in the Mongolian non-banking financial sector is growing year after year [4]. Thus, it is proposed to compare the internal variables/factors of individuals through the economic variables.

Numerous research studies deal with the issue of researching the impacts of economic factors on consumer credit risk determinants [53,54,55]. Changes in economic variables, such as (1) personal expenses including mortgage or rent payments, loans, insurance, daycare, tuition, and utilities; (2) personal incomes including salaries, wages, and bonuses received from employment or self-employment, and rental receipts from real estate investments, have strong forecasting power for changes in the consumer credit risks. The importance of considering income–expense differentials in the household and elaborating consumer credit is highlighted in [56]. In this study, we investigated the influence of economic factors on consumer credit risk in terms of psychological factors. The descriptions of the economic variables used in this study are as follows:

- Salary—What is your monthly income?
- Extra income—What is the amount of your extra income?
- Salary loan—Do you have a salary loan?
- NBFI loan—Do you repay loans at banks and NBFIs?
- Previous NBFI loan—Have you ever received loans from banks and NBFIs?
- Mortgage loan—Do you have a mortgage loan?
- Monthly payment—How much money do you spend on servicing loans each month?
- Real estate—Do you have an immovable property under your name?
- Real estate price—What is the current market price of your immovable property?
- Car—Do you own a car?
- Car price—What is the current market price of your car?
- Life year—How long have you lived at your current address?

### 3.5. Measure

The findings presented in this study are part of broader research investigating the significance of the psychological indicators in credit risk assessment. For the purpose of the study, a structured questionnaire consisting of 111 closed-ended questions was designed (as provided in Supplementary File). The closed-ended type was considered appropriate since it requires little time and effort to be completed (discussed in Section 3.2). In total, 1118 borrowers took part in the study.

Once the questionnaires were collected, the data were analyzed with statistical methods. Logistic regression was employed to estimate the parameters of a logistic model. According to [57], logistic regression is a statistical method for analyzing a dataset in which there are one or more independent variables that determine an outcome. The outcome is estimated with a dichotomous variable (in which there are only two possible outcomes). In logistic regression, the dependent variable is binary or dichotomous, meaning that it only contains data coded as 1 (such as “True”, “Yes”) or 0 (“False”, “No”). If the dependent variable is continuous, it can be dichotomized at some logically meaningful cut point. The goal of logistic regression is to find the best-fitting and logically reasonable model to describe the relationship between an outcome (dependent or response variable) and a set of independent (predictor or explanatory) variables [57]. Moreover, unlike classic linear regression where the parameters are computed using the least-squares method, logistic regression estimates the parameters using the likelihood ratio. In this way, the response variable (predicted) is a function of the likelihood that a particular observation (individual) will be in one of the two categories of the dichotomy.

In the present study, since the dependent variable was binary (dichotomous), the function of the binary logistic regression was

(1) $$f\left(Z\right)=\frac{e^{z}}{1+e^{z}}=\frac{1}{1+e^{-z}}$$

where Z is the input variable and f(Z) is its outcome. One of the advantages of this function is that the input variable takes positive and negative values, whereas the outcome f(Z) ranges between 0 and 1. More analytically, variable Z represents the combined influence of a set of variables, while f(Z) defines the likelihood of a specific outcome resulting from this action. In addition, variable Z expresses the measure of the overall contribution of all participating independent variables to the model and is defined as

(2) $$Z=\beta_{0}+\beta_{1}X_{1}+\beta_{2}X_{2}+\cdots +\beta_{k}X_{k}$$

where $\beta_{0}$ is the intercept of the regression line, and $\beta_{i}$ is the coefficient of the independent variables, expressing the contribution of each variable.

When a coefficient takes a positive value, the explanatory variable increases the likelihood of a successful outcome (i.e., the realization of the event). Conversely, a negative coefficient value means that the variable decreases the likelihood of the outcome. In addition, a high value of the coefficient would signify that the independent variable significantly affects the likelihood of the realization of the event, whereas a low value would denote a small effect of the independent variable on the likelihood of having the relevant result. Following the majority of the prior literature, we refer to the model as being constructed with the outcome f(Z) taking the value of 1 for failed (risky customers) and 0 for non-failed (non-risky customers).

## 4. Results

### 4.1. Factor Analysis

For construct validation of psychological questionnaires, we used confirmatory factor analysis (CFA). CFA is a multivariate statistical procedure used to test how well the measured variables represent the number of constructs.

Firstly, we checked if factor analysis was feasible. The Bartlett sphericity test checks the inter-correlation between manifest variables, which compares the observed correlation matrix and the identity matrix. If factor analysis is an appropriate method to use, the correlation matrix and the identity matrix will not be the same, and the test will be significant. Bartlett’s sphericity test ($\chi^{2}=\;$10,722.543; *p* < 0.001) affirmed that the matrix was not an identity matrix to ensure the factor analysis’s feasibility. The Bartlett sphericity test based on our data produced a significant p-value of 0.0.

Secondly, we checked whether it was appropriate to use the manifest variables for factor analysis. The test involves the computation of the proportion of variance among the manifest variables. The Kaiser–Meyer–Olkin (KMO) sampling adequacy measure was used. Both results indicate that it was possible to extract factors from the matrix of observed correlations. The KMO values range between 0 and 1, and a proportion under 0.6 would suggest that the dataset is inappropriate for factor analysis. Our data were still appropriate with the KMO test at 0.688.

Table 5 presents the standardized coefficients of correlation between factors and those between variables and factors. The resulting factor loading for each variable reached high values. Except for one item (Q119; 1 of 21) from the effective economic decision-making subscale, only one item (Q205; 1 of 16) from the self-control subscale, two items (Q305 and Q307; 2 of 18) from the conscientiousness subscale, and two items (Q403 and Q409; 2 of 16) from the selflessness and giving attitude subscale, the indicators had loadings that exceeded 0.70. No item found from the attitude towards the money subscale had loadings lower than 0.70 (0 of 25), but six items were found from the neuroticism subscale (Q503, Q507, Q508, Q510, Q512, Q514, and Q515; 6 of 15). All indicators were statistically significant, with *p* < 0.01. The lowest loading was for Q508 (I can have fun even when I’m alone), with a value of 0.586 in the subscale neuroticism. The highest loading was for Q113 (I am taking the necessary steps to achieve my financial goals), with a value of 0.882 in the subscale effective economic decision-making. The obtained results support the six-factor structure proposed for the instrument and, thus, provide strong evidence of construct validity for the model. Please see the details of all questions in Supplementary File.

### 4.2. Analysis of Psychological Variables

Logistic regression analysis was used in testing the correlation between individual characteristics and debt repayment tendency. The independent variables of the analysis included 6 psychological variables, while the dependent variable was the loan default statistics of borrowers. The regression analysis established the correlation between the 6 psychological factors and loan default with the inclusion of a control variable. For the data used in the research, the longer days in arrear represented positive values, while higher scores in psychological factors represented negative values. The score of psychometrics which measured psychological factors has inverse correlation with the loan default. Thus, a positive correlation between the regression values represents the proof of the research hypothesis. See Table 6 for the regression result.

The analysis revealed that it was slightly significant with effective economic decision-making 0.096 (*p* < 0.001), self-control 0.053 (*p* < 0.001), conscientiousness 0.054 (*p* < 0.001), selflessness and giving attitude 0.068 (*p* < 0.001), neuroticism −0.062 (*p* > 0.05), and attitude to money 0.104 (*p* < 0.001). Variables such as effective economic decision-making, self-control, conscientiousness, selflessness and giving attitude, and attitude towards money had high significance and played a role in predicting credit risks. In other words, these psychological factors can serve as a basis for timely repayment of the loan. On the other hand, neuroticism was insignificant.

Hypothesis H1 suggests that the ability to make efficient economic decisions is significant in predicting the borrower’s risk. Hypothesis H2 suggests that self-control has a significant impact on timely debt payment. Hypothesis H3 suggests that a conscientious personality significantly reduces the risk of having overdue credit. Hypothesis H4 suggests that a charitable attitude and selfless personality significantly/positively affect the loan risk. Hypothesis H6 suggests that a positive attitude towards money positively affects the loan risk. These hypotheses were supported. In contrast, Hypothesis H5 was not supported. A summary of the hypotheses, proposed relationships, and testing results is presented in Table 7.

### 4.3. Analysis on Psychological Variables through Economic Factors

In addition, we compared the psychological variables through the traditional economic factors such as salary, extra income, salary loan, NBFI loan, previous NBFI loan, mortgage loan, monthly payment, real estate, real estate price, car, car price, life year, as well as different age and gender groups.

One of the reasons that households apply for loans may be a precondition for long-standing, sustainable livelihood. In general, income grows at the beginning of active economic activities and shrinks after retirement, so that debt is a tool to flatten the expenditure to be used throughout one’s life. From the lender’s perspective, the debt service capacity is analyzed in line with one’s income. However, poor management of one’s income can lead to default regardless of the amount of income, which was proven in the report mentioned above [4]. Besides income, personal responsibilities and discipline are proven to play a role in debt service, a hypothesis presented through this research, and analyzed through logistic regression. The data for economic variables in this research were based on the inputs made by the loan applicants. Those variables represent the living conditions and financial capacities.

As shown in Table 8, the financial variables were differently associated with all six psychological variables. Among the personality traits, neuroticism was negatively associated with economic variables. In contrast, the other five psychological variables were positively associated with the economic variables.

For example, effective financial decision-making, self-control, and conscientiousness were strongly significant (*p* < 0.001) when respondent’s salary level was between 800,000MNT and 1,200,000MNT.Whereas selflessness and giving attitude were only significant when respondent’s salary level was lower than 800,000MNT or between 1,200,000MNT and 2,000,000MNT.Attitude to money was significant when only the salary level was between 1,200,000MNT and 2,000,000MNT. Self-control was strongly significant when salary level was lower than 1,200,000MNT. Effective economic decision-making was highly, positively correlated with salary level. However, extra income was more related to two psychological factors: decision-making and self-monitoring. Generally, in terms of economic indicators, the availability of any loan type was highly correlated with the psychological factors except neuroticism and attitude to money. In other words, responsible borrowers, as determined by psychological factors, have a previous credit experience. Effective economic decision-making and self-control were very strongly significant (*p* < 0.001) among the people who pay between 300,000MNT and 500,000MNT and lower than 300,000MNT per month, respectively. People who did not own real estate had a higher dependence on psychological factors than people who did not own real estate. On the other hand, people with cars had a higher dependence on psychological factors than people without cars. That means that there were more people with cars who did not have real estate among microcredit clients. People who lived in the same place for more than three years were highly dependent on effective economic decision-making, conscientiousness, and selflessness and giving attitude. People in their 30s and 50s had more dependency on effective financial decision-making, conscientiousness, selflessness and giving attitude, while self-control had more dependency on people in their 20s and 30s, and attitude toward money in their 30s had more dependency. While effective economic decision-making and selflessness and giving attitude were independent of gender, self-control and attitude to money were more dependent on women, and conscientiousness was more dependent on men.

## 5. Findings and Discussion

**Figure 2:**
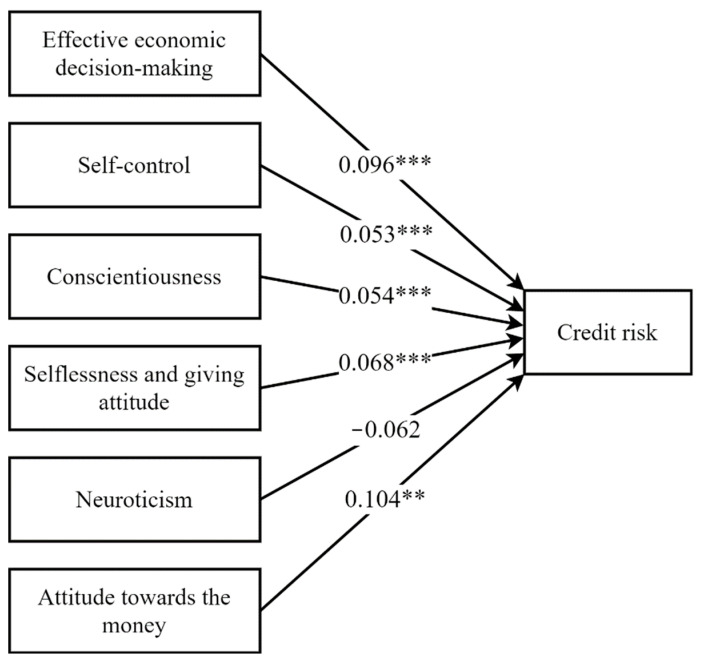
Result of research model and hypotheses.

This study of borrowers with different types of loan repayment behaviors uncovered the following interesting features. Firstly, based on this research, it is established that psychology, especially certain personal traits, plays a significant role in identifying the debt service capabilities of individuals, as presented in Hypotheses H1–H4 and H6. As shown in Figure 2, positive results of psychometric tests in the areas of effective financial decision-making, self-control, conscientiousness, selflessness and giving attitude, and attitude toward money enables debt access possibilities of individuals. Secondly, our findings suggest that there is no significant difference in economic and demographic characteristics between responsible borrowers and debtors. A study on whether economic indicators can predict debt service capabilities revealed that high income, ownership of real estate or cars, having a salary and consumer loans, and servicing other large loans each month have no significance. From our results, we conclude that the difference in loan repayment behavior is caused mostly by personal and internal characteristics rather than by demographic and financial characteristics.

These findings support previous research that psychological factors can predict loan repayment [1,9,21]. In addition, the fact that neuroticism was not related to loan repayment [21] is associated with the results of this study. According to the findings, one of the main characteristics of regular loan payers is “conscientiousness”, and some researchers found that conscientious individuals can accumulate savings dedicated to their health and pension [36], have better credit history [12], and have the tendency to be organized and dependable, show self-discipline, act dutifully, and aim for achievement [1]. Additionally, respondents who scored high on “effective financial decision-making” describe their financial decision making with the statement of “I can keep expenses on budget”. This is similar to findings of Maria A. Gagarina and Anna A. Shantseva (2017) [21], who studied the socio-psychological peculiarities and level of financial literacy of Russian debtors and found that debtors with low rational debt behavior made impulsive decisions. Another common characteristic of regular payers, referring to “self-control”, has also become evident by the findings of this research. In contrast, the results show that risky borrowers are characterized with “selfishness and taking attitude” and “negative attitude towards money”. People that are unable to spend money on others and tend to hold grudges, we may assume, lack the soul to pay the loan back. By benefiting from the literature, characteristics of an individual’s credit risk framed by findings of this research were refined into more comprehensive “themes” to reveal influential factors on repayment behavior.

Finally, in the case of Mongolia, this result entails that assessing personal traits and psychological factors of borrowers rather than economic indicators is more effective in ensuring reliability and risk-averseness of the loans issued.

To this end, the following dimensions are suggested as a result of our findings:

- As shown in H1, protecting oneself from acting on a whim and making a budget and purchase decisions are based on logic and analysis and essentially enables one to practice proper money management and to be left with sufficient money for the loan repayment. This result supports the findings that rational decision-making is a prominent characteristic of responsible borrowers [1].
- H2 reveals that poor self-control leads to overspending and unnecessary purchases, thus leaving no room for servicing one’s loans. This result supports the idea that lack of self-control and a low level of financial literacy are positively correlated with defaults on consumer loans and self-reports of excessive debt burden [34].
- As reflected in H3, a conscientious person has better financial control, holds savings, and ensures timely debt servicing. This result supports some research findings which state that responsible borrowers have a higher level of conscientiousness and rational debt behavior than risky borrowers [21]. Additionally, researchers stated that conscientious individuals are responsible, attentive, careful, persistent, orderly, and planful, while those people who score low on this trait are irresponsible, unreliable, careless, and distractible [58].
- As shown in H4, a generous and selfless individual with a mission to serve others tends to repay loans. This result supports the theory which says that people who are unable to spend money on others tend to lack soul to pay the loan back [38].
- As reflected in H5, the hypothesis of the psychological factor, neuroticism, was not supported. As compared against the other indicators, neuroticism is volatile and cannot serve as an indicator to predict a consistent outcome. This finding supports the result of a previous survey [21]. However, this result is in disagreement with Nyhus and Webley (2001) [12], who found that emotional instability (i.e., neuroticism) is a predictor of loan default. The reason for this result needs to be re-examined, and we assume that there are differences in national characteristics.
- As demonstrated in H6, the possibility to service loans on time is increased when one neither worships nor denies money. Conversely, worshipping or fearing money has a propensity to lead one to either deny using money or emotionally spend to accommodate one’s psychological issues. This leaves one to have no money to spend on servicing loans.

## 6. Conclusions, Implications and Limitations

Predicting credit risk in microloan services plays a key role in the further development of this sector. While it is difficult to incorporate a psychometric analysis in credit risk assessment, it is considered to be helpful in expanding the borrower pool and improving the trust between the lender and borrower, as compared to financial indicators. Combining technology with the science of psychology to issue loans to borrowers, regardless of their employment status and age, based on trust, enables those who are denied loans accesses at other financial institutions. One of main results of this research contributes to opening loan access to those unable to obtain loans so that tangible and intangible assets will be generated to the livelihoods of the people; unfair competition is reduced, social accessibility is increased, poverty is alleviated, and sustainable development is achieved.

Our research demonstrates that psychological indicators play a more significant role in predicting debt servicing than economic indicators. An important issue related to the psychological questionnaire is how to verify whether borrowers will pay back a loan. In addressing this issue, we confirmed the factor analysis by CFA. Thus, the article provided a valid and reliable questionnaire for assessing psychological factors causing loan repayment behavior in Mongolia. Regression analysis on economic and psychological indicators revealed that internal factors of individuals are more effective in the prediction of loan default than material factors (economic factors). In other words, the shortcomings related to the usage of only economic factors can be remedied with the inclusion of psychological factors. Findings displayed as main characteristics of responsible borrowers include effective financial decision-making, self-control, conscientiousness and selflessness and giving (charitable) attitude, and positive money attitude, while irresponsibility, irrational financial decision making, negative attitude towards money, low self-control, and selfishness and taking attitude characterized high-risk borrowers. In comparison with the other determinants, neuroticism cannot serve as an indicator to predict credit risk, and we suggest that further investigation is needed.

The empirical evidence exists to demonstrate that there are many possible causes of the increasing level of debt worldwide [9]. The novelty of this research is that it enables further research angles in assessing individual behaviors and internal factors within the scope of psychology. In Mongolia, there is no prior study that focused on psychometric analysis alone. Analogous research is also rare at the international level too. Therefore, it is believed that the research will make contributions to the fields of psychology, behavioral economy, and economic sciences. Nevertheless, conducting research that combines financial standings with psychological factors is not easy. Going forward, further time and effort will be required in carrying out empirical research to prove the theoretical hypothesis.

The introduction of this methodology into the market enables the start of creating a first-ever, large-scale database on behaviors, characteristics, and lifestyles of Mongolians. This database has a far-reaching social contribution and may serve as a research-based resource that enables decision-makers to issue policies that cater to the needs of the public. With respect to practical contributions, this study helps to improve the understanding of behavioral and psychological factors that may lead to indebtedness and help financial institutions to build a reliable credit scoring model.

The limitation of our research compared to international research is that we paid little attention to the internal consistency and correlations of the psychological factors, which is mainly because of the scarcity of previous studies. One of the psychological factors was assessed as insignificant. Thus, data quality will be further improved to ensure the consistency of psychological factors. In addition, the research sample included customers only from the capital city of Mongolia, Ulaanbaatar, and this is a limitation because individuals from other areas may have different results depending on the environment, lifestyle, and characteristics. Moreover, the identification of loan repayment discrepancies covered only microlending customers through the mobile application, and it is possible to further expand the sample coverage. Future research may conduct a comparative study involving users and customers of other banks and non-bank financial institutions, as well as other countries, not only within the mobile database of mobile lending applications, and to re-examine the hypotheses made in this study.

## Figures and Tables

**Table 1:** Test developing methods.

Test Developing Method 1 [51]	Test Developing Method 2 [52]
(1) Establish an expert committee. (2) Identify dimensionality of construct. (3) Determine questionnaire format. (4) Determine item format. (5) Item development. (6) Determine questionnaire length. (7) Review and revise initial item pool. (8) Pilot testing. (9) Reliability/Validity check. (10) Subsequent validation.	Construct definition, specification of test need, test structure. Overall planning. Item development. Construct definition. Item generation: theory versus sampling. Item review. Piloting of items. Scale construction-factor analysis and Item Response Theory (IRT). Reliability. Validation. Test scoring and norming. Test specification. Implementation and testing. Technical Manual.

**Table 2:** Cronbach’s alpha values for scales.

Subscales	Number of Items	Cronbach’s Alpha
EDM	21	0.913
SCR	16	0.763
CON	18	0.698
SGA	16	0.694
NRT	15	0.677
ATM	25	0.873

EDM—Effective financial decision—making; SCR—Self-control; CON—Conscientiousness; SGA—Selflessness and a giving attitude; NRT—Neuroticism; ATM—Attitude toward money.

**Table 3:** Questionnaire examples.

Hypothesis	Question	Answer	Point
Effective financial Decision-Making			
H1	I can keep expenses on budget	No	−2
		b. Not sure	−1
		c. Likely yes	1
		d. Yes	2
	2. If we don’t have money to pay, it’s better not to buy	No	−2
		b. Not sure	−1
		c. Likely yes	1
		d. Yes	2
Self-control			
H2	I have a hard time breaking bad habits	Accept the proposal	−2
		b. I will think about it	−1
		c. Delay accepting the proposal	1
		d. Refuse from the proposal	2
	2. When you feel healthy before the end of treatment, what do you do?	Stop having medicine	−2
		b. Visit the doctor, after a short interruption	−1
		c. Continue having medicine to the end	1
		d. Visit the doctor after finishing the medicine	2
Conscientiousness			
H3	When you must attend an important meeting, you.	I will simply have a rest. Everything will be alright.	−2
		b. Despite my worries, I can take a good rest.	−1
		c. I will sleep after verifying essential things.	1
		d. I can’t sleep well because of my worries.	2
	2. In which case will you start cleaning the table?	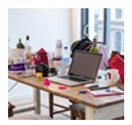	−2
		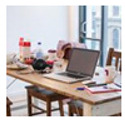	−1
		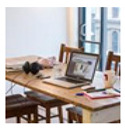	1
		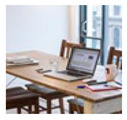	2
Selflessness and giving (charitable) attitude			
H4	I find it difficult to do work that is not specified in the job description	Definitely	−2
		b. Likely yes	−1
		c. Rarely	1
		d. Never	2
	2. Do you like to give presents?	Never	−2
		b. Only if I have to. Personally, I don’t feel any happiness from it	−1
		c. Yes, I like to give gifts to my friends and loved ones	1
		d. Gifts are given only when needed	2
Neuroticism			
H5	I worry about many things.	Exactly	−2
		b. Yes	−1
		c. No	1
		d. Never	2
	2. I am afraid of making a mistake in front of the public.	Never	−2
		b. No	−1
		c. Yes	1
		d. Exactly	2
Attitude towards the money			
H6	I spend money on valuable things only	No	−2
		b. Not sure	−1
		c. Likely yes	1
		d. Yes	2
	2. I avoid talking about money	Always	−2
		b. Mostly	−1
		c. Seldom	1
		d. Never	2

**Table 4:** Descriptive statistics of dependent and independent variables.

Variables	Mean	Std. Dev.	Min	25%	50%	75%	Max
Dependent variable							
Credit Risk	0.75	0.43	0	1	1	1	1
Psychological variables							
EDM	3.38	3.31	−7	1.5	4	6	9.5
SCR	3.33	4.83	−10	0	2.5	7.5	10
CON	3.65	4.53	−10	0	5	7.5	10
SGA	2.46	3.77	−7	−0.5	3.5	6	7
NRT	−0.23	2.17	−4	−2	0	1	4
ATM	1.20	1.89	−4	0	1	3	4

**Table 5:** Factor loadings for the six-factor model, including correlations between error terms (details in Supplementary File).

Items	Loading	$R^{2}$	Items	Loading	$R^{2}$	Items	Loading	$R^{2}$
**EDM**			**SCR**			**CON**		
Q101	0.748	0.559	Q201	0.705	0.497	Q301	0.811	0.658
Q102	0.754	0.568	Q202	0.733	0.537	Q302	0.779	0.607
Q103	0.791	0.626	Q203	0.722	0.521	Q303	0.809	0.655
Q104	0.779	0.606	Q204	0.790	0.624	Q304	0.768	0.590
Q105	0.808	0.654	Q205	0.664	0.441	Q305	0.689	0.475
Q106	0.796	0.634	Q206	0.756	0.572	Q306	0.715	0.512
Q107	0.785	0.616	Q207	0.731	0.534	Q307	0.694	0.481
Q108	0.862	0.743	Q208	0.750	0.562	Q308	0.806	0.650
Q109	0.781	0.610	Q209	0.758	0.574	Q309	0.793	0.629
Q110	0.864	0.746	Q210	0.717	0.514	Q310	0.738	0.545
Q111	0.809	0.654	Q211	0.736	0.541	Q311	0.778	0.606
Q112	0.799	0.639	Q212	0.770	0.593	Q312	0.770	0.593
Q113	0.882	0.777	Q213	0.768	0.590	Q313	0.782	0.611
Q114	0.776	0.602	Q214	0.752	0.566	Q314	0.789	0.623
Q115	0.877	0.769	Q215	0.720	0.519	Q315	0.744	0.553
Q116	0.771	0.595	Q216	0.712	0.506	Q316	0.775	0.601
Q117	0.797	0.635				Q317	0.716	0.513
Q118	0.777	0.604				Q318	0.787	0.619
Q119	0.671	0.451						
Q120	0.796	0.633						
Q121	0.786	0.617						
SGA			NRT			ATM		
Q401	0.722	0.522	Q501	0.745	0.555	Q601	0.801	0.641
Q402	0.742	0.550	Q502	0.823	0.677	Q602	0.836	0.699
Q403	0.650	0.422	Q503	0.624	0.390	Q603	0.808	0.653
Q404	0.766	0.586	Q504	0.825	0.681	Q604	0.767	0.588
Q405	0.754	0.568	Q505	0.737	0.544	Q605	0.840	0.705
Q406	0.775	0.601	Q506	0.818	0.670	Q606	0.786	0.618
Q407	0.771	0.595	Q507	0.681	0.463	Q607	0.804	0.647
Q408	0.743	0.552	Q508	0.586	0.343	Q608	0.777	0.604
Q409	0.652	0.425	Q509	0.720	0.518	Q609	0.856	0.733
Q410	0.786	0.618	Q510	0.695	0.484	Q610	0.773	0.598
Q411	0.779	0.606	Q511	0.790	0.624	Q611	0.819	0.671
Q412	0.728	0.530	Q512	0.599	0.359	Q612	0.805	0.648
Q413	0.729	0.532	Q513	0.788	0.621	Q613	0.850	0.723
Q414	0.715	0.511	Q514	0.620	0.384	Q614	0.850	0.722
Q415	0.731	0.535	Q515	0.649	0.422	Q615	0.854	0.729
Q416	0.732	0.536				Q616	0.860	0.739
						Q617	0.851	0.723
						Q618	0.850	0.722
						Q619	0.825	0.681
						Q620	0.852	0.727
						Q621	0.844	0.713
						Q622	0.846	0.716
						Q623	0.859	0.738
						Q624	0.841	0.708
						Q625	0.856	0.733

Note: $R^{2}$ = Percentage of variance explained.

**Table 6:** Logistic regression results of psychological variables.

Variable	B	S.E.	Wald	Significance	Low Conf.	High Conf.
EDM	0.096	0.017	5.670	0.000	0.062	0.129
SCR	0.053	0.013	4.125	0.000	0.028	0.078
CON	0.054	0.013	4.135	0.000	0.029	0.08
SGA	0.068	0.016	4.155	0.000	0.036	0.100
NRT	−0.062	0.032	−1.955	0.051	−0.125	0.000
ATM	0.104	0.034	3.067	0.002	0.037	0.170

**Table 7:** Summary of hypothesis testing results.

Hypothesis	Relationship	Result
**H1**	The ability to make efficient economic decisions is significant in predicting the borrower’s risk.	Supported
**H2**	Self-control has a significant impact on timely debt payment.	Supported
**H3**	A conscientious personality significantly reduces the risk of having overdue credit.	Supported
**H4**	Charitable attitude and selfless personality significantly/positively affect the loan risk.	Supported
**H5**	Neurosis significantly affects loan defaults.	Not supported
**H6**	A positive attitude towards money positively affects the loan risk.	Supported

**Table 8:** Summary of hypothesis testing results.

Category	EDM	SCR	CON	SGA	NRT	ATM
Salary ≤ 800 K	0.082 *	0.058 *	0.026	0.091 **	−0.12	0.094
800 K < Salary ≤ 1.2 M	0.122 ***	0.063 **	0.103 ***	0.036	−0.071	0.054
1.2 M < Salary ≤ 2 M	0.069 *	0.033	0.036	0.081 **	0.001	0.137 *
Salary > 2 M	0.135	0.102	0.040	0.138	−0.178	0.304
Extra income = 0	0.051	0.019	0.092 ***	0.124 ***	−0.066	0.230 **
Extra income ≤ 400 K	0.113 ***	0.055 **	0.044 *	0.044	−0.071	0.065
400 K < Extra income ≤ 800 K	0.125 ***	0.036	0.059 *	0.061	−0.028	0.032
Extra income > 800 K	0.089	0.17 ***	0.009	0.037	−0.167	0.245 *
Salary loan = No	0.101 ***	0.042 *	0.069 ***	0.059 **	−0.061	0.139 **
Salary loan = Yes	0.088 ***	0.068 ***	0.036	0.082 ***	−0.065	0.057
NBFI loan = No	0.109 ***	0.052 *	0.055 *	0.061 *	−0.039	0.122 *
NBFI loan = Yes	0.087 ***	0.055 ***	0.054 **	0.071 ***	−0.076	0.094 *
Previous NBFI loan = No	0.100**	0.027	0.088 **	0.020	−0.017	0.159 *
Previous NBFI loan = Yes	0.095 ***	0.062 ***	0.043 **	0.082 ***	−0.081 *	0.090 *
Mortgage loan = No	0.067	0.060	0.102 *	0.067	−0.095	0.159
Mortgage loan = Yes	0.098 ***	0.053 ***	0.05 ***	0.067 ***	−0.061	0.100 **
Monthly payment = 0	0.091 **	0.013	0.067 **	0.055	−0.068	0.16 *
Monthly payment ≤ 300 K	0.107 **	0.086 ***	0.046	0.093 **	−0.15 *	0.062
300 K < Monthly payment ≤ 500 K	0.114 ***	0.072 **	0.055 *	0.049	−0.028	0.140 *
Monthly payment > 500 K	0.073 *	0.049	0.056 *	0.092 **	−0.013	0.018
Real estate = No	0.091 ***	0.052 ***	0.055 ***	0.062 ***	−0.078 *	0.105 **
Real estate = Yes	0.275 **	0.031	0.034	0.166 *	0.397	0.170
Real estate price ≤ 20 M	0.090 ***	0.059 ***	0.058 ***	0.087 ***	−0.126 **	0.106 *
20 M < Real estate price ≤ 50 M	0.130 ***	0.045	0.039	0.023	0.084	0.148
50 M < Real estate price ≤ 80 M	0.028	0.043	0.076 *	0.055	−0.061	0.172
Real estate price > 80 M	0.159 **	0.033	0.044	0.093	−0.027	0.050
Car = No	0.027	0.073	0.080	0.006	−0.205	0.067
Car = Yes	0.102 ***	0.052 ***	0.051 ***	0.074 ***	−0.048	0.112 **
Car price ≤ 8 M	0.089 ***	0.059 **	0.056 **	0.060 **	−0.137 **	0.064
8 M < Car price ≤ 13 M	0.095 **	0.031	0.039	0.111 ***	−0.028	0.144 *
13 M < Car price ≤ 18 M	0.097	0.040	0.079	0.042	0.082	0.135
Car price > 18 M	0.212 *	0.114	0.054	−0.093	0.017	0.253
Life year ≤ 3	0.071	0.098 *	0.066	0.031	0.003	0.212 *
3 < Life year ≤ 6	0.074 *	0.038	0.068 **	0.061 *	−0.023	0.083
6 < Life year ≤ 10	0.15 ***	0.039	0.000	0.083*	−0.057	0.139
Life year > 10	0.095 ***	0.059 **	0.062 **	0.074 **	−0.091	0.075
Age ≤ 22	0.505	0.319	−0.028	−0.001	0.192	−0.659
22 < Age ≤ 30	0.033	0.112 **	0.058	0.071	−0.183 *	0.158
30 < Age ≤ 50	0.106 ***	0.030 *	0.053 ***	0.074 ***	−0.047	0.088 *
Age > 50	0.102 *	0.111 **	0.050	0.014	0.032	0.196 *
Sex = Female	0.106 ***	0.078 ***	0.037	0.058 *	−0.091	0.114 *
Sex = Male	0.088 ***	0.028	0.073 ***	0.076 ***	−0.044	0.091

Note: * means *p* < 0.05, ** means *p* < 0.01, *** means *p* < 0.001.

## Data Availability

The authors will share data from the study upon reasonable request to the corresponding author.

## Supplementary Materials

The following are available online at https://www.mdpi.com/article/10.3390/bs11040047/s1.
