# Supplementary material for: Effect of Psychological Factors on Credit Risk: A Case Study of the Microlending Service in Mongolia
Source: Behav Sci (Basel). 2021 Apr 5;11(4):47. doi: 10.3390/bs11040047 (PMC8067141; doi:10.3390/bs11040047)
Supplement: Supplementary file 1 [file behavsci-11-00047-s001.pdf]

Appendix A. List of Psychological Questions

An expert revision was obtained on the developed questionnaire. Necessary corrections were made based on the experts' conclusions. As shown in Table A1, the revised questionnaire itself contains 111 questions, which contain hypothesis codes, questions, answers, and points for corresponding answers.

Table A1. List of psychological questions.

Hypothesis	Question	Answer	Point
**Effective financial decision-making**			
H1	Q101. Which of the following would be the most annoying for you?	a. When I can't afford something I want to get	-2
		b. Credits that I didn't pay	-1
		c. Losing someone's trust	1
		d. Promises I couldn't keep	2
	Q102. What kind of person wouldn't be friends with? Someone …	a. A selfish person who never takes care of others	-2
		b. A person who offended me	-1
		c. A person who doesn't keep their promises	1
		d. A person who promises impossible things	2
	Q103. What kind of person do you prefer to be? Someone …	a. who never lies	-2
		b. who has many friends	-1
		c. who doesn’t hurt others	1
		d. who do what they can do	2
	Q104. In my view, having a debt is …	a. something makes me irritated	-2
		b. not something good	-1
		c. something solves my financial problems	1
		d. not problem for me because I can always repay	2
	Q105. What would be the reason when someone refused to lend you money?	a. They are so mean	-2
		b. Such a selfish person, not realizing my condition	-1
		c. Maybe they don't lend money to anyone	1
		d. It’s okay. Maybe they were not possible	2
	Q106. What would be your response when someone doesn’t give you the money they borrowed?	a. Usually I forget about things like that	-2
		b. Wait for them to give the money back	-1
		c. Do everything to get the money back	1

		d. Remind them to give the money back	2
	Q107. What would you do when you meet someone who hasn't given your valuable thing back? Choose one of the following	a. Yell at them and claim my belonging	-2
		b. Firstly greet and then claim my belonging	-1
		c. Don't say anything until they mentions about it	1
		d. Kindly ask my belonging	2
	Q108. I feel anger when I receive bills	a. Strongly agree	-2
		b. Agree	-1
		c. Disagree	1
		d. Strongly disagree	2
	Q109. I am afraid of financial loss	a. Strongly agree	-2
		b. Agree	-1
		c. Disagree	1
		d. Strongly disagree	2
	Q110. I saved some money for rainy days	a. No	-2
		b. Don't know	-1
		c. Maybe	1
		d. Yes	2
	Q111. When I am having a lot of money …	a. That never happens	-2
		b. I worry	-1
		c. I feel calm	1
		d. I think about saving it	2
	Q112. When I am having little money	a. I am used to it	-2
		b. I feel anger and insecure	-1
		c. I feel bit worry and then think about getting more money	1
		d. I don't worry because I can earn money again	2
	Q113. I am taking the necessary steps to achieve my financial goals.	a. Never	-2
		b. Rarely	-1

		c. Often	1
		d. Always	2
	Q114. I can keep expenses on budget	a. Yes	-2
		b. Maybe	-1
		c. Don't know	1
		d. No	2
	Q115. I am independent person	a. No	-2
		b. Don't know	-1
		c. Maybe	1
		d. Yes	2
	Q116. Which of the following can influence the most when you decide the restaurant to have dinner?	a. Popular or not	-2
		b. Whether I have visited there before	-1
		c. Restaurant's location	1
		d. Affordability	2
	Q117. Does money change human attitude?	a. Yes definitely	-2
		b. Less money leads to negative attitude	-1
		c. Money strengthens a person's character	1
		d. One should not be controlled by money	2
	Q118. Which of the following can influence the most when you consider the quality of the product you want to get?	a. Higher price	-2
		b. Experienced before	-1
		c. Whether it is widely used or not	1
		d. Affordable price	2
	Q119.Consider you have agreed to meet your friend at 18:00. What time would you arrive?	a. 18:10	-2
		b. 18:00	-1
		c. 17:50	2
	Q120. Consider you can’t go to your friend’s birthday party. How do you tell a friend about it?	a. I will not tell	-2
		b. Text on social media	-1
		c. Text sms	1
		d. I will call	2
	Q121. If we don’t have money to pay, it's better not to buy	a. No	-2
		b. Not sure	-1
		c. Likely yes	1
		d. Yes	2
**Self-control**			

H2	Q201. If you have a reason to change yourself, what would be the reason?	a.	to lead my team	-2
		b.	to get job	-1
		c.	for my family	1
		d.	to impress someone I like	2
	Q202. What advice would you give to current high school students?	a.	Create the best memories, because childhood will never be found again	-2
		b.	Study hard	-1
		c.	It is better to learn from generation before you	1
		d.	Being kindness is more important than being knowledgeable	2
	Q203. Which of the following is the most embarrassing?	a.	Can't do the things that other can do	-2
		b.	Mistakes I made	-1
		c.	Others thinking I am weak	1
		d.	Lack of skills	2
	Q204. You have a big exam tomorrow but you haven't prepared well enough. What would you do?	a.	Cheat on exam	-2
		b.	Lie that I am sick and don't take exam	-1
		c.	Ask help from others	1
		d.	Even I didn't prepare, just go and take the exam	2
	Q205. How satisfied are you about your life?	a.	1-3	-2
		b.	4-6	-1
		c.	7-8	1
		d.	9-10	2
	Q206. An old acquaintance offered you a high-paying job. But you have already planned your career. What will be your decision?	a.	I accept the offer	-2
		b.	I reconsider the offer	-1
		c.	I postpone the offer	1
		d.	I reject the offer	2
	Q207. You are in a rush and need to tell urgent information to your colleague when he is on the phone. What would be your response?	a.	Wait until he finish talking	-2
		b.	I have no time so I will whisper	-1
		c.	I directly tell him cause it is urgent	1

		d.	Wait for a while and gesture to tell something important	2
	Q208. After a busy working day, you came home and wanted to watch your favorite TV show. But it is almost sleep time and TV can irritate other family members. What would you do?	a.	Just keep watching the show	-2
		b.	Volume down and continue watching the show	-1
		c.	Ask others whether it is fine to watch TV or not	1
		d.	Turn the TV off	2
	Q209. Which of the following might be the most important to you?	a.	Making others happy	-2
		b.	Not hurting others	-1
		c.	Not disturbing others	1
		d.	Respecting others	2
	Q210. Which of the following might be the biggest fear for you?	a.	To be hated	-2
		b.	To lose	-1
		c.	To be irresponsible	1
		d.	To be rejected	2
	Q211. For me, being dumped is	a.	It is okay. It will be better when time goes by	-2
		b.	Not a big deal	-1
		c.	Would be hard but I can go through it	1
		d.	Would be the hardest thing and it is my biggest fear	2
	Q212. When you feel healthier before the end of treatment, what would you do?	a.	Stop having medicine	-2
		b.	Visit the doctor, after a short interruption	-1
		c.	Continue having medicine to the end	1
		d.	Visit the doctor after finishing the medicine	2
	Q213. I think i need to be more disciplined	a.	Strongly agree	-2
		b.	Agree	-1
		c.	Disagree	1
		d.	Strongly disagree	2
	Q214. Which one does help you to relax more?	a.	Smoking a cigarette	-2
		b.	A piece of cake	-1
		c.	Seeing a doctor	1
		d.	Meditation	2
	Q215. How do you describe the	a.	I work extra hours	-2

	balance of your work and personal life?	b. Sometimes i work extra hours	-1
		c. I enjoy personal life more than work	1
		d. I have a good work and personal life balance	2
	Q216. I have had a hard time breaking bad habits	a. Yes	-2
		b. Maybe	-1
		c. Don't know	1
		d. No	2
**Conscientiousness**			
H3	Q301.Have you ever not gone to work because you were fed up?	a. Yes	-2
		b. Maybe	-1
		c. Not exactly	1
		d. Never	2
	Q302. On average, how many hours a day does a team member who does not need to use the Internet surf the internet?	a. 0-15 minutes	-2
		b. 15-30 minutes	-1
		c. 30-60 minutes	1
		d. 60-90 minutes	2
	Q303. Have you ever promised something when you are emotional	a. Yes, I have forgotten the promise	-2
		b. That happened few times	-1
		c. Yes, but I regret about it	1
		d. I always be careful with making promises	2
	Q304. What is the most important thing you have learned from your parents?	a. Education	-2
		b. Manner	-1
		c. Responsibility	1
		d. Patience	2
	Q305. From whom do you usually hide your irresponsibility?	a. From myself	-2
		b. From my family	-1
		c. From my colleague	1
		d. From my friends	2
	Q306. You have to share 12 dollars with a stranger. There will be the following options. Which one do you	a. I get 8 dollars and a stranger gets 4 dollars	-2
		b. I get 6 dollars and a stranger gets 6 dollars	-1

	prefer?	c. I get 5 dollars and a stranger gets 7 dollars	1
		d. Decide by stranger's suggestion	2
	Q307. I have a bad memories about losing others' trust	a. Never	-2
		b. Maybe that happened 1-3 months ago	-1
		c. 2-3 years ago happened to me	1
		d. A year ago that happened	2
	Q308. You are at the neighbour's house to babysit their child, and he is asleep. Your neighbours will arrive in two hours but you are bored, what would you do?	a. Go and see the bedroom	-2
		b. Watch TV in the living room	-1
		c. Turn the computer on and surf internet	1
		d. Wash the dishes	2
	Q309. When you need to do something you don't like:	a. Leaving immediately	-2
		b. Get angry	-1
		c. Do it somehow	1
		d. It's normal to happen this kind of things	2
	Q310. Which of the following do you put aside the most?	a. Take care of others	-2
		b. Daily planning	-1
		c. Make time for myself	1
		d. Utility bills	2
	Q311. What percentage of your work is usually completed on time?	a. 0-24%	-2
		b. 25-49%	-1
		c. 75-100%	1
		d. 50-74%	2
	Q312. What do you do when you are stressed?	a. Become quiet	-2
		b. Focus on work	-1
		c. Talk	1
		d. Do what I like	2
	Q313. Which of the following payments do you usually delay?	a. Loan	-2
		b. Utility bills	-1
		c. Telecommunication fees	1
		d. Tuition fee	2
	Q314. You find that a letter addressed to someone has fallen to the ground. What would you do?	a. Will open the letter and read it	-2
		b. Leave it where it was. Because it doesn't matter to me	-1
		c. Will not read the letter, deliver it to the addressee	1
		d. Will tear the letter	2
	Q315.When you must attend an important meeting, you:	a. I will simply have a rest. Everything will be alright.	-2
		b. Despite my worries, I can take a good rest.	-1
		c. I will sleep after verifying essential things.	1
		d. I can't sleep well because of my worries.	2
	Q316. Once I start something, I finish it	a. No	-2
		b. Don't know	-1
		c. Maybe	1
		d. Yes	2

	Q317. What do you like most about your current job?	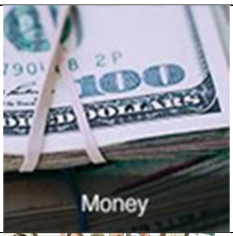	-2
		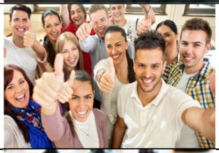	-1
		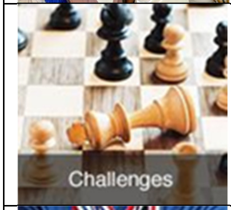	1
		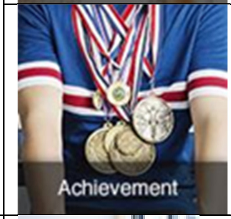	2
	Q318. In which case will you start cleaning the table?	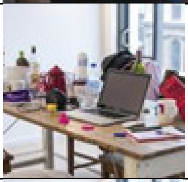	-2
		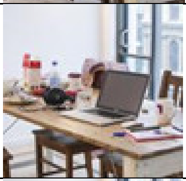	-1
		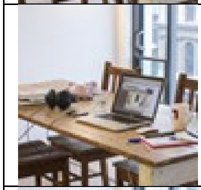	1
		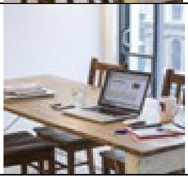	2
**Selflessness and giving (charitable) attitude**			
H4	Q401. Which of the following can disturb the family atmosphere from your perspective?	a. Rude attitude and communication	-2
		b. Not having fun	-1
		c. Not talking	1
		d. Not making time for each other	2
	Q402. Which is more important to you?	a. My efforts	-2
		b. Being calm	-1

		c. Making time for myself	1
		d. Having no family problems	2
	Q403. I spend .... hours a day with my family	a. Up to 1	-2
		b. 3-4	-1
		c. 1-2	1
		d. 2-3	2
	Q404. People say I am a family oriented person	a. Never	-2
		b. No	-1
		c. Yes	1
		d. Exactly	2
	Q405. Where do you like to be the most?	a. Everywhere is the same	-2
		b. Outdoors, in a cozy environment	-1
		c. At work	1
		d. At home	2
	Q406. When I feel sorry for others, I share what I have.	a. Never	-2
		b. No	-1
		c. Yes	1
		d. Exactly	2
	Q407. When you were a child, how familiar were you with your household's financial management	a. Strongly disagree	-2
		b. Disagree	-1
		c. Agree	1
		d. Strongly agree	2
	Q408. It is difficult to say no to my manager if they give an extra work.	a. Never	-2
		b. No	-1
		c. Yes	1
		d. Exactly	2
	Q409. Which purchase would you prefer?	a. Things I need	-2
		b. Things I want	-1
		c. Things my loved ones wants	1
		d. Things my family or co-worker needs	2
	Q410. I find it difficult doing the tasks which is not specified in my job description.	a. Often	-2
		b. Always	-1
		c. Rarely	1
		d. Never	2
	Q411. I'm not the person who trying to being agreeable with everyone	a. Yes	-2
		b. Maybe	-1
		c. Don't know	1
		d. No	2
	Q412. Do you like to give presents?	a. Never	-2
		b. Give present if I have to, unless don't feel happiness from giving a present to others	-1
		c. I like to give gifts to my friends and family	1
		d. Always happy to give presents	2
	Q413. There is a conflict about money in our family	a. Once and more in a week	-2
		b. Once in two weeks	-1
		c. Once in a quarter	1
		d. Once in a month	2
	Q414. You were traveling in a train with three people. The book you were reading was in a very interesting part.	a. Turn on the lights and read the book silently.	-2
		b. I apologize and leave the light on	-1

	The lights went out in the evening, and you wanted to read your book, but you would disturb others. What would you do:	c. Ask if the light can be turned on, and if not, leave it off	1
		d. Leave the light off	2
	Q415. Which of the following does matter to you the most?	a. Your room	-2
		b. Your house or flat	-1
		c. Your neighbourhood	1
		d. Your city	2
	Q416. The success of others inspires me	a. Never	-2
		b. No	-1
		c. Yes	1
		d. Exactly	2
**Neuroticism**			
H5	Q501. I worry about many things.	a. Exactly	-2
		b. Yes	-1
		c. No	1
		d. Never	2
	Q502.When something gets dirty, I feel uncomfortable	a. Never	-2
		b. I am not, because I will clean it later	-1
		c. When I am in public	1
		d. Often	2
	Q503. I regret the days I didn't go to work.	a. Strongly agree	-2
		b. Agree	-1
		c. Disagree	1
		d. Strongly disagree	2
	Q504. I eat nutritious food.	a. Never	-2
		b. Rarely	-1
		c. Always	1
		d. Often	2
	Q505. I try to change my behavior to suit the situation.	a. Never	-2
		b. Rarely	-1
		c. Often	1
		d. Always	2
	Q506. It's hard for me to control my impulsiveness.	a. Always	-2
		b. Often	-1
		c. Rarely	1
		d. Never	2
	Q507. I feel like I've taken on too much responsibility	a. Never	-2
		b. Rarely	-1
		c. Often	1
		d. Always	2
	Q508. I can have fun even when I'm alone.	a. Always	-2
		b. Often	-1
		c. Rarely	1
		d. Never	2
	Q509. I can refuse my order if it takes much difficulty to waitress.	a. Often	-2
		b. Always	-1
		c. Rarely	1
		d. Never	2
	Q510. I don't like facing troubles	a. Never	-2
		b. Rarely	-1

		c. Often	1
		d. Always	2
	Q511. I don't want to think about the future	a. Strongly agree	-2
		b. Agree	-1
		c. Disagree	1
		d. Strongly disagree	2
	Q512. I'm afraid that someone will downgrade me	a. Never	-2
		b. No	-1
		c. Yes	1
		d. Exactly	2
	Q513. I am very afraid of making mistakes in front of others	a. Never	-2
		b. No	-1
		c. Yes	1
		d. Exactly	2
	Q514. Which one is more similar to your work desk?	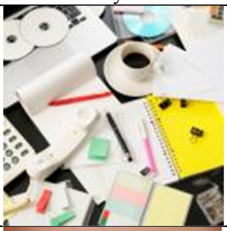	-1
		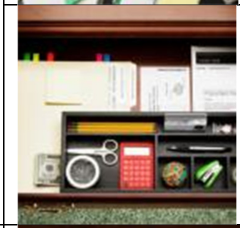	1
	Q515. If you could write down all the mistakes you've made in your life, which one would it be?	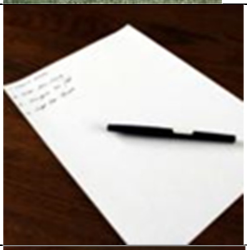	-2
		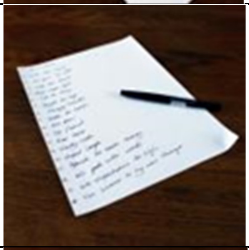	-1
		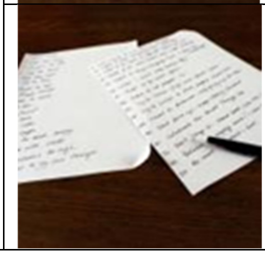	1

		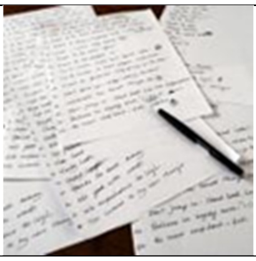	2
**Attitude towards the money**			
H6	Q601. I try not to think about money because I'm stressed out	a. Strongly agree	-2
		b. Agree	-1
		c. Disagree	1
		d. Strongly disagree	2
	Q602. It is not okay having more money than you need	a. Strongly agree	-2
		b. Agree	-1
		c. Disagree	1
		d. Strongly disagree	2
	Q603. Although I can afford it, I don't buy new things too often.	a. Strongly disagree	-2
		b. Disagree	-1
		c. Agree	1
		d. Strongly agree	2
	Q604. I can't throw away some things even I don't need them	a. Strongly agree	-2
		b. Agree	-1
		c. Disagree	1
		d. Strongly disagree	2
	Q605. My financial situation make me feel safe and secure	a. Strongly disagree	-2
		b. Disagree	-1
		c. Agree	1
		d. Strongly agree	2
	Q606. My family resents me for working too hard for money	a. Strongly agree	-2
		b. Agree	-1
		c. Disagree	1
		d. Strongly disagree	2
	Q607. I try not to think about my financial situation	a. Strongly agree	-2
		b. Agree	-1
		c. Disagree	1
		d. Strongly disagree	2
	Q608. Money is for saving, not for spending	a. Strongly agree	-2
		b. Agree	-1
		c. Disagree	1
		d. Strongly disagree	2
	Q609. It is important to save money for things that may happen in the future	a. Strongly disagree	-2
		b. Disagree	-1
		c. Agree	1
		d. Strongly agree	2
	Q610. It's not easy for me to reduce impulsive buying	a. Strongly agree	-2
		b. Agree	-1
		c. Disagree	1
		d. Strongly disagree	2

	Q611. I often buy more than I can afford	a. Strongly agree	-2
		b. Agree	-1
		c. Disagree	1
		d. Strongly disagree	2
	Q612. At the end of the month, I run out of money due to the credit payment and expenses	a. Strongly agree	-2
		b. Agree	-1
		c. Disagree	1
		d. Strongly disagree	2
	Q613. I get anxious every time I look at my bank account balance	a. Strongly agree	-2
		b. Agree	-1
		c. Disagree	1
		d. Strongly disagree	2
	Q614. I have a problem suppressing my desire to buy what I like	a. Always	-2
		b. Often	-1
		c. Rarely	1
		d. Never	2
	Q615. I constantly follow my "budget"	a. Never	-2
		b. Rarely	-1
		c. Often	1
		d. Always	2
	Q616. It is difficult for me to follow financial plan	a. Always	-2
		b. Often	-1
		c. Rarely	1
		d. Never	2
	Q617. I economize for the future	a. Never	-2
		b. Rarely	-1
		c. Often	1
		d. Always	2
	Q618. I spend money on valuable things only	a. No	-2
		b. Not sure	-1
		c. Likely yes	1
		d. Yes	2
	Q619. When I was a child, my family usually asked for my opinion when buying something new	a. Strongly disagree	-2
		b. Disagree	-1
		c. Agree	1
		d. Strongly agree	2
	Q620. I carefully plan my spending before making a purchase	a. Strongly disagree	-2
		b. Disagree	-1
		c. Agree	1
		d. Strongly agree	2
	Q621. Others say I plan my finances well	a. No	-2
		b. Don't know	-1
		c. Maybe	1

		d. Yes	2
	Q622. I avoid talking about money.	a. Always	-2
		b. Mostly	-1
		c. Seldom	1
		d. Never	2
	Q623. I talk openly with my partner about money	a. Strongly disagree	-2
		b. Disagree	-1
		c. Agree	1
		d. Strongly agree	2
	Q624. In general, I'm the kind of person who spends more than I earn	a. Strongly agree	-2
		b. Agree	-1
		c. Disagree	1
		d. Strongly disagree	2
	Q625. Consider you will be rewarded with 500,000 MNT tomorrow. For what would you spend?	a. New clothes	-2
		b. Gift for other	-1
		c. Making repairs at home	1
		d. Savings	2
